# Modelling Trends of Climatic Variability and Malaria in Ghana Using Vector Autoregression

**DOI:** 10.1155/2018/6124321

**Published:** 2018-05-29

**Authors:** Sylvia Ankamah, Kaku S. Nokoe, Wahab A. Iddrisu

**Affiliations:** ^1^Department of Mathematics and Statistics, University of Energy and Natural Resources, P.O. Box 214, Sunyani, Ghana; ^2^Office of Deputy Vice Chancellor, Catholic University of Eastern Africa, P.O. Box 62157-00200, Nairobi, Kenya

## Abstract

Malaria is considered endemic in over hundred countries across the globe. Many cases of malaria and deaths due to malaria occur in Sub-Saharan Africa. The disease is of great public health concern since it affects people of all age groups more especially pregnant women and children because of their vulnerability. This study sought to use vector autoregression (VAR) models to model the impact of climatic variability on malaria. Monthly climatic data (rainfall, maximum temperature, and relative humidity) from 2010 to 2015 were obtained from the Ghana Meteorological Agency while data on malaria for the same period were obtained from the Ghana Health Service. Results of the Granger and instantaneous causality tests led to a conclusion that malaria is influenced by all three climatic variables. The impulse response analyses indicated that the highest positive effect of maximum temperature, relative humidity, and rainfall on malaria is observed in the months of September, March, and October, respectively. The decomposition of forecast variance indicates varying degree of malaria dependence on the climatic variables, with as high as 12.65% of the variability in the trend of malaria which has been explained by past innovations in maximum temperature alone. This is quite significant and therefore, policy-makers should not ignore temperature when formulating policies to address malaria.

## 1. Introduction

Malaria is considered endemic in over hundred countries across the globe and many cases of malaria and deaths due to malaria occur in Sub-Saharan Africa. The disease is of great public health concern since it affects people of all age groups more especially pregnant women and children because of their vulnerability [1, 2]. Malaria is caused by blood parasites transmitted from person to person through the bites of infected mosquitoes [3, 4]. In the absence of prompt and effective treatment, malaria remains a life-threatening disease and about 3.2 billion people, which are almost half of the world's population, are at risk of it [4]. As such, malaria has been described as an entrenched global health challenge. However, to a lesser extent, Asia, Latin America, the Middle East, and parts of Europe are also affected. Malaria can thus be said to affect people of all regions, both poor and rich [5].

Malaria is not only the burden of the health sector in various countries, but it also permeates every aspect of the social as well as economic lives of their people [6]. Indeed, attempts to control malaria in many Sub-Saharan African countries began decades ago. The aim was to reduce the malaria disease burden until it was no longer of public health significance. It was also recognized that malaria could not be controlled by the health sector alone; therefore multiple strategies were also put in place with other health related sectors. In view of this, a lot of interventions were put in place to help in the control of the deadly disease, but malaria continues to be the leading cause of morbidity (illness) in Ghana [7]. According to Rogers and Randolph [8], malaria is an extremely climate sensitive disease and Haines et al. [9] indicated that observations of short-term variations in climate or weather show that even small temperature increases and precipitation changes can result in measurable impacts on malaria. Current evidence, according to Githeko et al. [10], shows that interannual and interdecadal climate variability have influence on the epidemiology of vector borne diseases. At the continental level, this evidence has been assessed to help determine the possible consequences of climate change in the future. Climate change was defined by Palut and Canziani [11] in the Fourth Intergovernmental Panel on Climate Change (IPCC) Assessment Report as change in the state of the climate that can be identified (e.g., by using statistical test) by changes in mean and/or variability of its properties, and that persists for an extended period typically decades or longer. Climate change has therefore been a key issue around which global development policy is being framed especially in African countries and it is considered as one of the most serious environmental and human threats.

Adu-Prah and Tetteh [12] are of the opinion that generating relevant information on the role of temperature, rainfall, and humidity on malaria prevalence at different geographic scales is critical to efforts to combat the burden of prevalence. For effective climate change adaptation strategies which involves planning and the necessary implementation of disease control interventions appropriately, a better understanding of the relationship between climatic variables and malaria is needed.

Studies have been carried out that link climatic variability and incidence of malaria. For instance, Sena et al. [13] carried out studies which showed on the overall that monthly mean rainfall correlated positively but weakly at lag months two to four and relative humidity also correlated positively but weakly from zero to four months lag with occurrence of malaria in Southwest Ethiopia. Similarly, Weli and Efe [14] conducted a study that examined the effect of climate on malaria occurrence in Port Harcourt. The results showed that prevalence of malaria is significantly dependent on the increase in rainfall and decrease in temperature. Another study carried out by Omonjo et al. [15] indicated that an increase in air temperature and sea surface temperature was associated with monthly malaria occurrence in the derived savannah and humid forest zones at various percentages. Time series plots revealed that malaria cases are common to both zones of the state and it occurs throughout the year but more prevalent at the beginning of rainy season. Haque et al. [16] also carried out studies to determine the effect of climatic variability on malaria incidence in Bangladeshi Highlands. They however did not find any association between temperature, humidity, rainfall, and malaria cases.

Meanwhile, the results from a study by Asare and Amekudzi [17] to assess climate driven malaria variability indicated that the intra- and inter-agro ecological variability in terms of intensity and duration of malaria transmission are predominantly controlled by rainfall. Also, Darkoh et al. [18] investigated the effects of climatic variables on malaria incidence in Ghana. A time series analysis was carried out. From their analysis, they detected that mean minimum and maximum monthly temperatures lagged at three months were significant predictors of malaria. However, rainfall was found not to be significant. In a study carried out by Klutse et al. [19], the transition and coastal savannah zones of Ghana were chosen and used as study sites. Their results showed that maximum temperature was a better predictor of malaria trends than minimum temperature or precipitation especially in the transition zone. Krefis et al. [20] modelled the relationship between precipitation and malaria incidence in children from a Holoendemic Area in Ghana. They conducted a time series analysis using cross-correlation function and autoregressive modelling on weekly basis in two village clusters in the study site. They observed that the regression model showed that the level of rainfall predicted the malaria incidence after a time lag of nine weeks for both clusters. Further, the cross-correlation functions showed a strong association between rainfall and the malaria incidence one or two weeks later dependent on the village cluster. This implies that the past period values for the cross-correlation function are much shorter than the past period values for the regression models although both tests implied there is a relationship between rainfall and malaria incidence. Their results indicated that malaria incidence can be predicted directly by high-resolution (large amount of) precipitation data in highly endemic areas indicating that a strong temporal relationship between rainfall and incidence of malaria exists.

Within Ghana, there are variations in climatic conditions during any one season or period since the northern part is mostly savannah, the middle belt tropical forest, and the coastal area savannah. These differences will have direct links to mosquito habitats and therefore the transmission of malaria. Therefore, a knowledge of climatic variability and its influence on the incidence of malaria can be used to predict malaria occurrences and hence provide early warning system information to health administrators and decision- and policy-makers for the control of the disease. In relation to modelling malaria and climatic variability, the use of multivariate models, especially, vector autoregression models, is however limited. Little has been done in the area of developing multivariate models that combines climatic variability to predict the seasonal prevalence of malaria cases in the country and inform the development of warning systems. Most studies used univariate methods in predicting malaria incidence. This study will develop a multivariate model in the form:

(1) $$\begin{array}{c} Y_{t}=c+\underset{1}{\prod }Y_{t-1}+\underset{2}{\prod }Y_{t-2}+\ldots +\underset{p}{\prod }Y_{t-p}+\varepsilon_{t} \end{array}$$

where ∏_i_ are (n×n) coefficient matrices and *ε*_t_ is an (n×1) unobservable zero mean white noise vector process (serially uncorrelated or independent) with time invariant covariance matrix Σ.

The objective of this study is to model the trends of climatic variability and malaria in Ghana using vector autoregression.

## 2. Materials and Methods

### 2.1. Study Area

**Figure 1:**
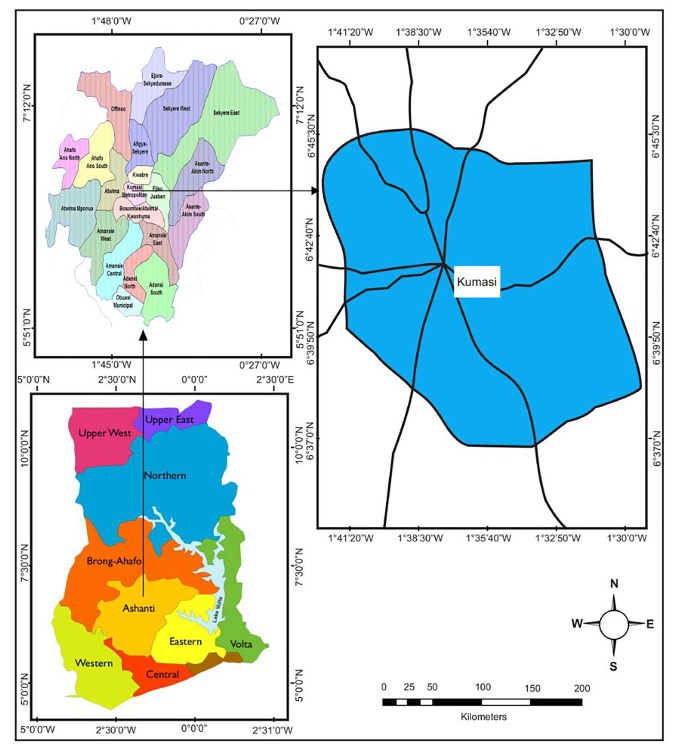
Map of Kumasi Metropolitan Area.

The study area used in this study is Kumasi Metropolitan Area in Ashanti Region in Ghana. The Kumasi Metropolitan Area (Figure 1), the capital of the Ashanti Region, is a commercial and industrial area and is one of the 30 administrative districts (1 metropolitan, 7 municipal, and 22 ordinary districts) in the Ashanti Region. The Metropolis lies in the transitional forest zone in south-central Ghana. It is the second largest most populous city in the country and located between Latitude 6°35′ and 6°47′N and Longitude 1°30′ and 1°43′W. It is elevated at 250 to 300 meters above sea level and covers an area of approximately 214.3 square kilometres.

The study area falls within tropical forest zone in Ghana and is dominated by the maritime air from the south and this results in two rainy seasons throughout the year. The first begins in April and ends in July whereas the second begins in September and ends in November. These two rainy seasons are both followed by the dry season.

### 2.2. Data Collection

The quantitative data consisted basically of secondary data from the Ghana Meteorological Agency and Health Service. The meteorological variables (2010-2015) include rainfall, maximum temperature, and relative humidity and were on mean monthly basis. The malaria data were also obtained based on monthly cases for the years 2010-2015 in the Kumasi Metropolitan Area.

### 2.3. Vector Autoregression Model

Vector autoregression (VAR) model developed by Sims [21] has been extensively used in econometrics for the analysis of multivariate time series. It is a natural extension of the univariate autoregressive model to dynamic multivariate time series and has superior forecast ability compared with those of univariate time series model. It also determines how each endogenous variable responds over time to a shock in its own value and in every other variable and allows the data to guide the researcher.

The basic form of the VAR model of order p suggested by Sims [21] has the form

(2) $$\begin{array}{c} y_{t}=A_{1}y_{t-2}+A_{2}y_{t-2}+\cdots +A_{p}y_{t-p}+{CD}_{t}+u_{t} \end{array}$$

where *y*_*t*_ = (*y*_1*t*_, *y*_2*t*_,…,*y*_*kt*_)′ is a vector of K observable endogenous variables.

For this study, *y*_*t*_ = (*Malaria*_*t*_, *TMax*_*t*_, *Rain*_*t*_, *RH*_*t*_)′, where* Malaria* represent the number of malaria cases per month,* Tmax* represent the maximum temperature,* Rain* is the amount of rainfall (mm), and* RH* is the relative humidity (%). *D*_*t*_ contains all deterministic variables which may consist of a constant, linear trend or seasonal dummy variables. *u*_*t*_ is a K-dimensional unobservable zero mean white noise process with positive definite covariance matrix *E*(*u*_*t*_*u*_*t*_′) = Σ_*u*_. *A*_*i*_ and C are parameter matrices of suitable dimension on which you can impose various restrictions.

The parameters in the model are estimated by generalised least squares.

### 2.4. Augmented Dickey Fuller Test

The Augmented Dickey Fuller (ADF) test is a unit root test for stationarity. The null hypothesis for the ADF test is that there is a unit root while the alternative hypothesis differs slightly according to the equation used. The simplest alternative hypothesis is that the time series is stationary. Unit roots can cause unpredictable results in time series analysis.

### 2.5. Optimal Lag Length Selection Criteria

For a range of lag orders *n* the individual equations of the system are estimated by OLS. The optimal lag order is selected by minimizing one of the following Information Criteria:

(3) Akaike Information Criterion,AICn=log⁡det⁡∑u^n+2TnK2

(4) Hannan-Quinn criterion,HQn=log⁡det⁡∑u^n+2log⁡log⁡TTnK2

(5) Schwarz Criterion,SCn=log⁡det⁡∑u^n+log⁡TTnK2

(6) Final Prediction Error criterion,FPEn=T+n∗T−n∗Kdet⁡∑u^n

where $\hat{\sum_{u}}(n)$ is estimated by $T^{-1}\sum_{t=1}^{T}\hat{u_{t}}{\hat{u_{t}}}^{\prime }$ and *n*^*∗*^ is the total number of parameters in each equation of the model when *n* is the lag order of the endogenous variables.

### 2.6. Structural Analysis

Despite the fact that VAR coefficients capture the anticipated impact of a variable, there are often a lot of coefficients to interpret. It is usually more common to examine the model's residuals which represent unforeseen contemporaneous events. The next subsections provide relatively nontechnical explanations of some of the common techniques used for structural analysis of VAR models.

#### 2.6.1. Causality Analysis

Both the Granger-causality and instantaneous causality were investigated. For both tests, the vector of endogenous variables is divided into two subvectors, *y*_1*t*_  *and*  *y*_2*t*_, with dimensions *K*_1_  *and*  *K*_2_, respectively, so that *K* = *K*_1_ + *K*_2_. The subvector *y*_1*t*_ is said to be Granger-causal for *y*_2*t*_ if the past of *y*_1*t*_ significantly helps predicting the future of *y*_2*t*_ via the past of *y*_1*t*_ alone [22]. For testing this property, a model of the form

(7) $$\begin{array}{c} \left[\begin{array}{c} y_{1t}\\ y_{2t} \end{array}\right]=\overset{p}{\underset{i=1}{\sum }}\left[\begin{array}{cc} \alpha_{11,i} & \alpha_{12,i}\\ \alpha_{21,i} & \alpha_{22,i} \end{array}\right]\left[\begin{array}{c} y_{1,t-i}\\ y_{2,t-i} \end{array}\right]+CD_{t}+\left[\begin{array}{c} u_{1t}\\ u_{2t} \end{array}\right] \end{array}$$

is considered. In this model setup, *y*_1*t*_ is not Granger-causal for *y*_2*t*_ if and only if

(8) $$\begin{array}{c} \alpha_{21,i}=0,\;i=1,2,\ldots ,p. \end{array}$$

Therefore, this null hypothesis is tested against the alternative that at least one of the *α*_21,*i*_ is nonzero. An F-test statistic which is distributed as *F*(*pK*_1_*K*_2_, *KT* − *n*^*∗*^) is used for testing the restrictions. Here *n*^*∗*^ is the total number of parameters in the system including the parameters of the deterministic term [23]. The role of *y*_1*t*_  *and*  *y*_2*t*_ can be reversed to test Granger-causality from *y*_2*t*_ to *y*_1*t*_.

Instantaneous causality is characterized by nonzero correlation of *u*_1*t*_  *and*  *u*_2*t*_. Thus, the null hypothesis

(9) $$\begin{array}{c} H_{0}:E\left(\;u_{1t}u_{2t}^{\prime }\;\right)=0 \end{array}$$

is tested against the alternative of nonzero covariance between the two error vectors in testing for instantaneous causality. A Wald test statistic is used to test this hypothesis.

#### 2.6.2. Impulse Response Analysis

In impulse response analysis, the exogenous and deterministic variables are treated as fixed and may therefore be dropped from the system. The adjusted endogenous variables are now denoted by *y*_*t*_. If the process *y*_*t*_ is stationary (I(0)), it has a Wold moving average (MA) representation

(10) $$\begin{array}{c} y_{t}=\Phi_{0}u_{t}+\Phi_{1}u_{t-1}+\Phi_{2}u_{t-2}+\cdots , \end{array}$$

where Φ_0_ = *I*_*K*_ and the Φ_*s*_ can be computed recursively as

(11) $$\begin{array}{c} \Phi_{s}=\overset{s}{\underset{j=1}{\sum }}\Phi_{s-j}A_{j},\;s=1,2,\ldots , \end{array}$$

with Φ_0_ = *I*_*K*_ and A_*j*_ = 0  *for*  *j* > *p*. The coefficients of this representation may be interpreted as reflecting the responses to impulses hitting the system. The (*i*, *j*)*th* elements of the matrices Φ_*s*_, regarded as a function of *s*, trace out the expected response of *y*_*i*,*t*+*s*_ to a unit change in *y*_*jt*_ holding constant all past values of *y*_*t*_.

#### 2.6.3. Forecast Error Variance Decomposition

Denoting the (*i*, *j*)*th* element of the orthogonalized impulse response coefficient matrix Ψ_*n*_ by *ψ*_*ij*,*n*_, the variance of the forecast error (*y*_*k*,*T*+*h*_ − *y*_*k*,*T*+*h*∣*T*_) is

(12) σk2h=∑n=0h−1ψk1,n2+⋯+ψkK,n2=∑j=1Kψkj,02+⋯+ψkj,h−12.

The term (*ψ*_*kj*,0_^2^ + ⋯+*ψ*_*kj*,*h*−1_^2^) is interpreted as the contribution of variable *j* to the *h*-step forecast error variance of variable *k*. Dividing the above terms by *σ*_*k*_^2^(*h*) gives the percentage contribution of variable *j* to the *h*-step forecast error variance of variable *k*,

(13) $$\begin{array}{c} w_{kj}\left(\;h\;\right)=\frac{\left(\psi_{kj,0}^{2}+\cdots +\psi_{kj,h-1}^{2}\right)}{\sigma_{k}^{2}\left(h\right)} \end{array}$$

### 2.7. Mean Absolute Percentage Error

The accuracy of forecasts generated by the fitted model was evaluated using mean absolute percentage error (MAPE) values.

(14) $$\begin{array}{c} MAPE=\left(\;\frac{1}{T}\overset{T}{\underset{t=1}{\sum }}\left|\;\frac{F_{t}-Y_{t}}{Y_{t}}\;\right|\;\right)100 \end{array}$$

where *F*_*t*_ is the forecasted value and *Y*_*t*_ is the observed value.

## 3. Results

### 3.1. Description of the Data

**Figure 2:**
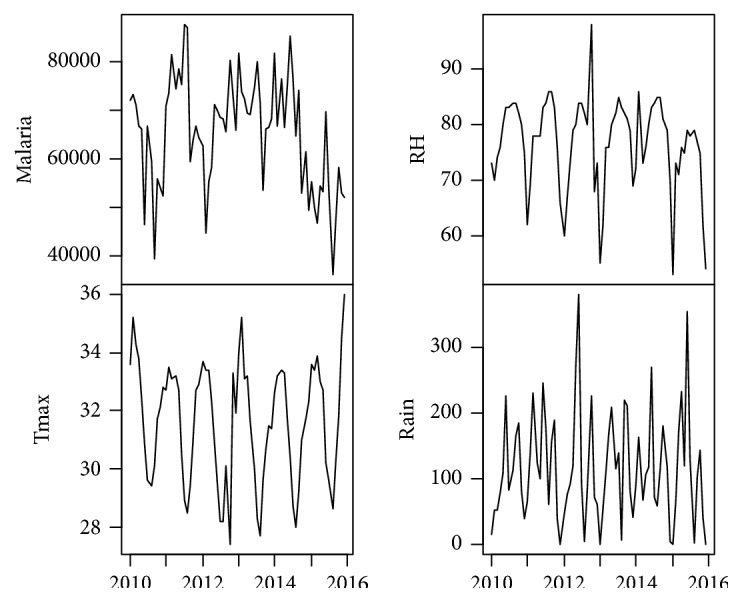
Time series plot of malaria and the climatic variables.

**Figure 3:**
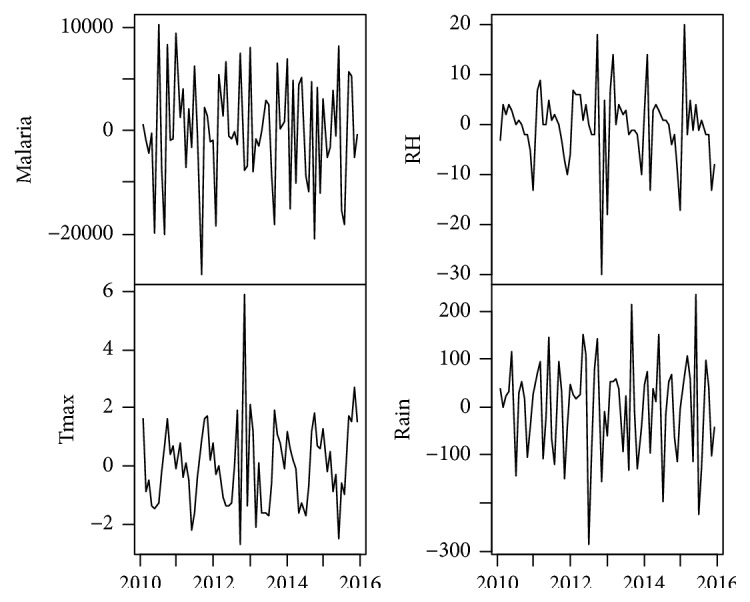
Time series plot of the first difference of malaria and climatic variables.

The data for this study comprises time series of monthly cases of malaria for the study area and some climatic variables including rainfall (Rain), maximum temperature (Tmax), and relative humidity (RH). The climatic data were obtained from the Ghana Meteorological Agency for January 2010 to December 2015 while the malaria data were obtained from the Ghana Health Service, also for January 2010 to December 2015.  Table 1 shows the descriptive statistics for the four variables considered. The minimum, maximum, and average values of malaria are 36047, 87765, and 65318, respectively. For the climatic variables, whereas the minimum, maximum, and average values of rainfall are 0.00mm, 379.80mm, and 115.26mm, respectively, the minimum, maximum, and average values of maximum temperature are 27.40°C, 36.00°C, and 31.62°C, respectively, while those of relative humidity are 53.00%, 98.00%, and 76.62%, respectively. The time series plots of the original data are presented in Figure 2 which indicates an obvious seasonality in both malaria and the climatic variables considered. The first difference of the data was obtained and plotted in Figure 3 which gives an indication of stationarity in both malaria and the climatic variables.

Each variable in the differenced data was tested for the presence of a unit root using a test suggested by Dickey and Fuller [24]. The test, presented in Tables 2(a) and 2(b), rejected the assumption of a unit root for all time series considered, implying that the relationships among the various variables analyzed below are not spurious.

### 3.2. VAR Estimation

A VAR model of the monthly malaria and climatic variables was estimated with 12 lags for each variable in each equation. Each equation has 4x12 unrestricted coefficients plus one coefficient for a constant and one for a trend. The number of lags was chosen based on four tests: the Final Prediction Error (FPE) test [25], the Hannan Quinne (HQ) test [26], and the Information Criteria suggested by Akaike (AIC) [27] and by Schwarz (SC) [28]. All four tests indicated that as many as 12 lagged monthly values may be sufficient. A lag length of 12 ensures that all the dynamics in the data are captured and are used in this analysis (Table 3).

### 3.3. Granger and Instantaneous Causality Tests

The instantaneous and Granger-causality tests [22] are simple ways to ascertain whether a particular variable is affected by innovations in other variables. These tests indicate if innovations in one variable help forecast a one-step ahead figure in another variable. An important advantage of these tests is that they are unaffected by the ordering of the VAR system. The test statistics are summarized in Tables 4 and 5. Each column contains the values of F-statistics testing the marginal effect of inclusion of lagged values of the climatic variables in the row on malaria.

Both tests indicate that malaria is influenced by all three climatic variables (relative humidity, rainfall, and maximum temperature). An important caveat about the above F-Tests must be noted. Although they indicate whether other variables Granger-cause malaria, it is still possible that other variables can influence malaria through other equations in the system. For this reason, we turn to the decomposition of the variances of forecast errors.

### 3.4. Decomposition of Variance and Residual Correlations

**Figure 4:**
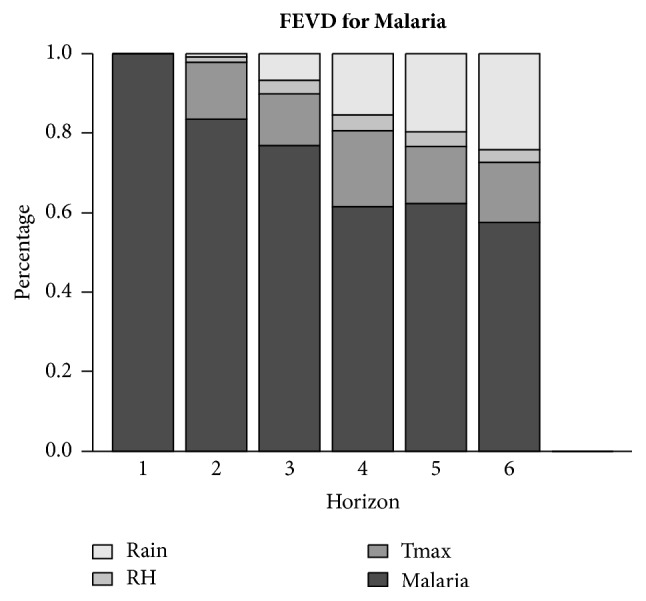
Forecast error variance decomposition.

Forecast error variance decomposition (FEVD) is popular in interpreting VAR models. Results for the FEVD for both malaria and the climatic variables are presented in Figure 4. The results reveal that on the average, while about 73.67% of the variability in the trend of malaria has been explained by past innovations in malaria cases, a significant proportion (about 12.65%) of the variability in the trend of malaria has been explained by past innovations in maximum temperature. Also, whereas about 11.10% of the variability in the trend of malaria has been explained by past innovations in rainfall figures, only 2.58% of the variability in the trend of malaria has been explained by past innovations in relative humidity.

### 3.5. Impulse Response

**Figure 5:**
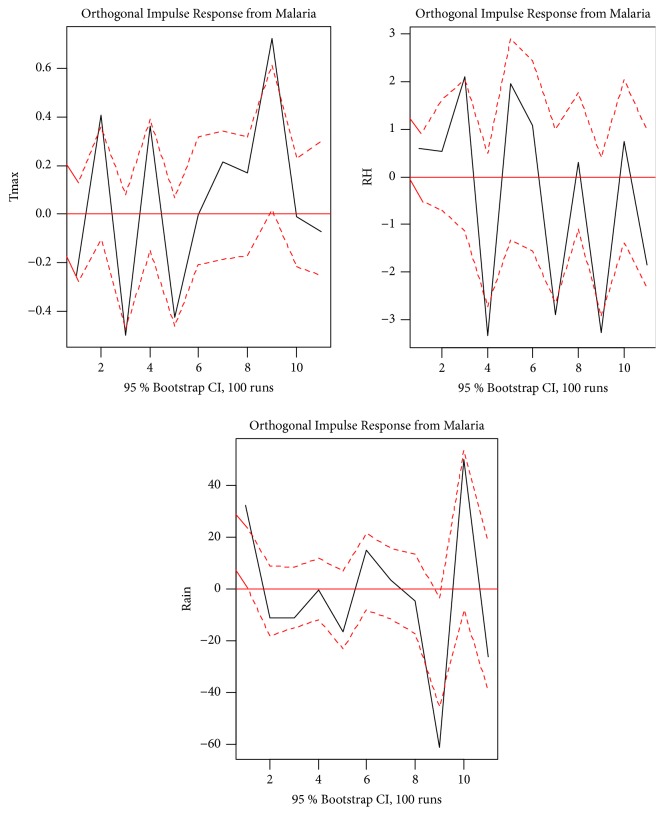
Impulse response analysis.

Impulse response analysis was utilized to analyze the dynamic interactions between malaria and the climatic variables of the VAR (12) process. The orthogonal impulse response of malaria to the climatic variables is presented in Figure 5. The response of malaria has an obvious fluctuation; there is a highest positive effect of maximum temperature on malaria in the ninth month (September) and lowest negative effect of maximum temperature on malaria in the third month (March). Also, the highest positive effect of relative humidity on malaria is observed in the third month (March) while the highest positive effect of rainfall on malaria is observed in the tenth month (October).

### 3.6. Malaria Forecast

**Figure 6:**
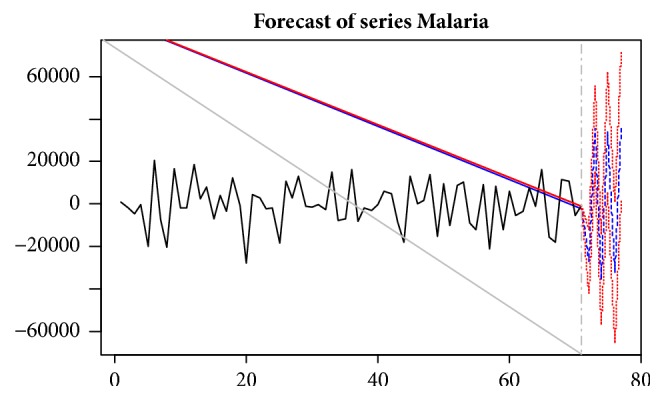
Forecast series of malaria.

The VAR (12) model developed can be used as a predictive model for making forecasts of future malaria cases. The forecasts for the differenced malaria cases for the first half of 2016 are presented in Figure 6 which is converted to the original level in Table 6. These forecasts indicate an increasing trend of malaria for the first half of 2016 in the Kumasi Metropolitan Area.

### 3.7. Forecast Accuracy

The mean absolute percentage error (MAPE) provides an indication of the average size of forecasting error expressed as a percentage of the relevant observed value irrespective of whether that forecasting error is positive or negative [29]. The MAPE for the fitted model is calculated to be 12.95% using (14) which is an indication of potentially very good forecasts by the fitted model [29].

## 4. Discussion

In this study, it was discovered that all three climatic variables influence malaria in the Kumasi Metropolitan Area. The highest positive effect of maximum temperature on malaria is in September, whereas the lowest negative effect is in March. Also, the highest positive effect of relative humidity on malaria is observed in March while the highest positive effect of rainfall on malaria is observed in October.

Further results indicate that on the average, while a greater percentage of the variability in the trend of malaria has been explained by past innovations in malaria cases, a significant proportion (about 12.65%) of the variability in the trend of malaria has been explained by past innovations in maximum temperature. Also, about 11.10% of the variability in the trend of malaria has been explained by past innovations in rainfall figures and only 2.58% of the variability in the trend of malaria has been explained by past innovations in relative humidity. This further indicates that the three climatic variables have varying effect on malaria with maximum temperature having a greater effect, followed by rainfall and then relative humidity. Therefore, modelling malaria and the climatic variables together will improve the forecast of malaria. These results are in line with studies conducted by Darkoh et al. [18] and Klutse et al. [19]. The results from the study conducted by Klutse et al. [19] showed that maximum temperature was a better predictor of malaria trends than minimum temperature or precipitation especially in the transition zone, whereas results obtained by Darkoh et al. [18] indicate that rainfall is not significant. However, minimum and maximum temperature are better predictors. Other studies include that of Adu-Prah and Tetteh [12] who discovered that temperature and relative humidity both have influence on malaria. However, the effect of rainfall was found to be less significant. Komen et al. [30] also discovered that temperature was more important in influencing malaria in the Limpopo District. They also discovered that rainfall has an influence on temperature and vice versa. However, the findings in this study contradict those obtained by Asare and Amekudzi [17]. Their results indicated that the intra- and inter-agro ecological variability in terms of intensity and duration of malaria transmission are predominantly controlled by rainfall. The variation in results obtained could be due to various reasons such as the study site chosen and the time frame of the study. The VAR (12) model developed can also be used as a predictive model for making forecasts of future malaria cases. The forecasted values obtained indicate an increasing trend of malaria for the first half of 2016 in the Kumasi Metropolitan Area.

One limitation of this study is the nonavailability of data over a longer period of time. For time series models, the rule of thumb is that one should have at least fifty (50) to sixty (60) data points but preferably more than hundred (100) observations [31]. It is therefore suggested that future studies in this area of interest should consider more than hundred data points.

## 5. Conclusions

This paper develops and estimates a vector autoregression (VAR) model of the monthly malaria cases and some important climatic variables including rainfall, maximum temperature, and relative humidity in the Kumasi Metropolitan Area. The model is used to investigate the dynamic linkages between malaria and climatic variability. The model is also used to simulate the responses of malaria to innovations in climatic variability.

Results of the Granger and instantaneous causality tests lead to a conclusion that malaria is influenced by all three climatic variables. The impulse response analyses indicate that the highest positive effect of maximum temperature, relative humidity, and rainfall on malaria is observed in the ninth, third, and tenth months, respectively. The decomposition of forecast variance indicates varying degree of malaria dependence on the climatic variables, with as high as 12.65% of the variability in the trend of malaria being explained by past innovations in maximum temperature alone. Results obtained from this study are useful for policy-makers as this will help come up with policies knowing the effects of climatic variability on malaria incidence in the Kumasi Metropolitan Area.

## Figures and Tables

**Table 1:** Descriptive statistics.

	Minimum	1st quartile	Median	Mean	3rd quartile	Maximum
Malaria	36047	55284	66803	65318	73325	87765
Tmax	27.40	30.07	31.85	31.62	33.23	36.00
RH	53.00	73.00	78.50	76.62	83.00	98.00
Rain	0.00	60.27	103.75	115.26	166.38	379.80

**Table:** (a) Augmented Dickey Fuller (ADF) unit root test for malaria and maximum temperature

	Malaria			Tmax		
	tau3	phi2	phi3	tau3	phi2	phi3
Test-statistic	-3.5848	4.4182	6.5665	-3.7926	5.0994	7.3618
1pct	-4.04	6.50	8.73	-4.04	6.50	8.73
5pct	-3.45	4.88	6.49	-3.45	4.88	6.49
10pct	-3.15	4.16	5.47	-3.15	4.16	5.47

**Table:** (b) Augmented Dickey Fuller (ADF) unit root test for relative humidity and rainfall

	RH			Rain		
	tau3	phi2	phi3	tau3	phi2	phi3
Test-statistic	-3.6268	4.5966	6.8736	-4.341	6.2939	9.4236
1pct	-4.04	6.50	8.73	-4.04	6.50	8.73
5pct	-3.45	4.88	6.49	-3.45	4.88	6.49
10pct	-3.15	4.16	5.47	-3.15	4.16	5.47

**Table 3:** Optimal lag length selection.

Lag	AIC	HQ	SC	FPE
1	3.222141e+01	3.255130e+01	3.306651e+01	9.881112e+13
2	3.172640e+01	3.227623e+01	3.313490e+01	6.086123e+13
3	3.162184e+01	3.239159e+01	3.359374e+01	5.613160e+13
4	3.092405e+01	3.191372e+01	3.345935e+01	2.917406e+13
5	3.052771e+01	3.173732e+01	3.362641e+01	2.106825e+13
6	3.008062e+01	3.151015e+01	3.374272e+01	1.501048e+13
7	2.988180e+01	3.153126e+01	3.410730e+01	1.441608e+13
8	2.973971e+01	3.160911e+01	3.452861e+01	1.569618e+13
9	2.981110e+01	3.190042e+01	3.516340e+01	2.329626e+13
10	2.893095e+01	3.124020e+01	3.484665e+01	1.537346e+13
11	2.725518e+01	2.978436e+01	3.373428e+01	5.713970e+12
12	2.544600e+01	2.819510e+01	3.248850e+01	2.750571e+12

**Table 4:** Granger causality tests.

Cause variable	Null hypothesis	F-value	p-value	Decision
RH	RH does not Granger-cause Malaria	2.2201	0.009492	Reject the null hypothesis
Rain	Rain does not Granger-cause Malaria	2.1121	0.01381	Reject the null hypothesis
Tmax	Tmax does not Granger-cause Malaria	4.9524	2.747e-06	Reject the null hypothesis

**Table 5:** Instantaneous causality tests.

Cause variable	Null hypothesis	Chi-squared-value	p-value	Decision
RH	No instantaneous causality between RH and Malaria	20.872	0.0001119	Reject the null hypothesis
Rain	No instantaneous causality between Rain and Malaria	20.374	0.000142	Reject the null hypothesis
Tmax	No instantaneous causality between Tmax and Malaria	21.795	7.198e-05	Reject the null hypothesis

**Table 6:** Malaria forecast for the first half of 2016.

Month	Forecast	Lower	Upper	CI
Jan	55450.59	36772.56	74128.62	18678.03
Feb	58612.25	37218.14	80006.35	21394.10
Mar	60632.76	38454.46	82811.05	22178.30
Apr	61812.94	39302.72	84323.15	22510.21
May	62505.33	39750.81	85259.85	22754.52
Jun	62937.90	39946.85	85928.94	22991.05
